# Molecular Evidence of Clonal *Salmonella* Enteritidis Persistence in Poultry Cold-Chain Environments Under Environmental Stress

**DOI:** 10.3390/foods14223943

**Published:** 2025-11-18

**Authors:** Khaled S. Gazi, Wafa A. Alshehri, Alhanouf M. Alkhammash, Nada Alqadri, Fayez Saeed Bahwerth, Roua S. Baty, Nahlah N. Albakri, Ashjan F. Khalel, Tariq Abdulmutaleb Alpakistany, Mohammad Melebari

**Affiliations:** 1 Department of Biology, Faculty of Science, Al-Baha University, Al-Baha 65779, Saudi Arabia; kgazi@bu.edu.sa; 2 Department of Biological Sciences, College of Science, University of Jeddah, Jeddah 23890, Saudi Arabia; 3 Molecular Biology Unit, Riyadh Municipality Central Area Labs, Riyadh 12282, Saudi Arabia; alhanoufalkhammash@hotmail.com; 4 Biology Department, Turabah University College, Taif University, Taif 21974, Saudi Arabia; naqadri@tu.edu.sa; 5 Makkah Health Cluster, Makkah 24245, Saudi Arabia; fbahuwayrith@moh.gov.sa; 6 Department of Biotechnology, College of Science, Taif University, P.O. Box 11099, Taif 21944, Saudi Arabia; rsbaty@tu.edu.sa; 7 Department of Biology, College of Science, Taibah University, Madinah 42353, Saudi Arabia; nbakri@taibahu.edu.sa; 8 Biology Department, University College of Aldarb, Jazan University, Jazan 89872, Saudi Arabia; akhalel@jazanu.edu.sa; 9 Mental Health Complex, Laboratory Department, Microbiology Section, Ministry of Health, Taif 26521, Saudi Arabia; talpakistany@moh.gov.sa

**Keywords:** *Salmonella enteritidis*, poultry, cold-chain, ISSR, RAPD, molecular typing, food safety, fingerprinting

## Abstract

Breakdown of cold-chain integrity drives the persistence of foodborne pathogens in poultry supply chains in warm, mountainous climates. This study used Al-Mandaq (Saudi Arabia) as a model to assess genetic diversity and contamination in bacteria from poultry storage units using 16S rRNA sequencing, VITEK 2, selective culturing, and ISSR/RAPD fingerprinting on 150 swabs. The *Salmonella enterica* complex comprised 15/29 isolates (51.7%), followed by *Escherichia* spp. 6/29 (20.7%) and *Bacillus* spp. 3/29 (10.3%). Five *Salmonella serovars* were identified: Enteritidis (8), Waycross (3), Minnesota (2), Typhimurium (1), and Dublin (1). *S.* Enteritidis accounted for 8/29 isolates (27.6%) and predominated among *Salmonella* in supermarket retail samples in Al-Mandaq. Combined ISSR and RAPD cluster analysis revealed highly clonal *S.* Enteritidis groupings, consistent with cross-contamination and prolonged survival in refrigeration equipment. In resource-limited settings, the combined ISSR and RAPD approach enhanced identification and differentiation of bacterial contamination sources within refrigeration equipment, providing superior strain-level discrimination compared to single-marker systems and improving epidemiological traceability of cross-contamination events. These results highlight the risk of clonal pathogen persistence in poultry cold-chain environments and the value of integrated molecular fingerprinting for surveillance in challenging climates.

## 1. Introduction

The consumption of poultry meat globally has undergone significant growth in recent decades, positioning itself as a predominant animal protein source on a worldwide scale [1]. According to current projections from the OECD-FAO Agricultural Outlook, global poultry consumption is anticipated to reach 173 million tonnes by the year 2034, constituting 62% of the additional meat consumed globally [2]. The rise in consumption underscores poultry’s status as the most economically accessible meat option globally and its increasing acceptance among consumers who are conscious of sustainability [3,4,5]. With the escalating demand for poultry products, the significance of safety and hygiene protocols in their handling, storage, and retailing has emerged as a critical issue in public health [6,7,8]. Poultry meat is intrinsically highly perishable and prone to microbial contamination attributable to its elevated moisture content, nutrient-rich composition, and the extensive handling it undergoes during processing, packaging, and commercial display [9,10]. The presence of nitrogenous compounds, lipids, carbohydrates, vitamins, along with its high water-holding capacity, fosters an optimal environment for microbial proliferation, thereby rendering poultry products particularly susceptible to contamination by pathogenic microorganisms [11,12].

Contamination risks persist throughout the poultry supply chain, but retail environments particularly supermarket refrigeration units represent critical control points where multiple risk factors converge [13,14]. Insufficient storage conditions and temperature fluctuations in retail settings substantially enhance the survival and growth of pathogenic and spoilage microorganisms, thereby posing substantial risks to consumer health and contributing to outbreaks of foodborne illnesses. Whereas previous studies have indicated that poultry meat is susceptible to contamination at various points along the food supply chain, ranging from the farm to the market; contamination may occur during processing, contact with facility equipment, interaction with food handlers, and exposure to environmental sources [15,16,17]. Refrigeration units employed for the storage of poultry in supermarkets are crucial for maintaining the integrity of the cold chain and preventing microbial proliferation [18,19]. Nonetheless, these refrigeration units can paradoxically serve as reservoirs and vectors for pathogenic bacteria if not properly sanitized [20]. These microorganisms not only contribute to meat spoilage but also possess significant antimicrobial resistance traits, thereby complicating the treatment of infections and posing a threat to global public health initiatives [21,22,23]. Surveillance of contamination in retail poultry storage remains limited, particularly in developing regions. Further conventional methods lack the resolution to distinguish related strains or trace contamination routes. Therefore, advanced surveillance approaches are urgently needed for strain-level detection and source attribution.

Molecular fingerprinting techniques represent a critical advancement in addressing these surveillance limitations. Random Amplified Polymorphic DNA (RAPD-PCR) and Inter-Simple Sequence Repeat (ISSR-PCR) assays facilitate the genotypic characterization of isolates and enable high-resolution strain differentiation [24,25]. These methods are particularly advantageous for tracking genetic diversity and examining relatedness among resistant strains. RAPD analysis generally uncovers 60–80% polymorphic bands contingent upon the bacterial species, whereas ISSR markers frequently exhibit a slightly superior discriminatory capacity, with polymorphism rates ranging from 70 to 80% [26,27].

In Saudi Arabia, the Saudi Food and Drug Authority (SFDA) is the main regulatory body overseeing food safety, largely through post-market surveillance and monitoring initiatives [28]. Nevertheless, the availability of microbiological data pertaining to poultry storage environments in retail contexts, particularly within smaller urban areas and non-metropolitan regions, remains scarce. The specific environmental challenges of warm, mountainous climates such as those present in the Al-Baha region create particular obstacles to maintaining cold-chain integrity and necessitate region-specific surveillance data to inform evidence-based interventions. Al-Mandaq, a city situated in the Al-Baha region, exemplifies such a locale where comprehensive microbial evaluations have not been extensively documented, despite the proliferation of retail food markets. This geographic and climatic context makes the region an ideal model system for evaluating the utility of integrated molecular fingerprinting approaches in surveillance and source tracking within challenging environments. The acquisition of data concerning microbial load, patterns of antimicrobial resistance, and the genetic variability of bacterial populations on poultry refrigeration surfaces offers critical insights for addressing regional food safety challenges. Within this framework, the current study investigated bacterial contamination present on refrigeration surfaces within Al-Mandaq supermarkets. The isolates were identified using both culture-based and molecular methodologies, while PCR-based techniques were employed to evaluate genetic diversity. Specifically, we applied an integrated ISSR and RAPD fingerprinting approach to provide enhanced strain-level discrimination and source tracking capabilities. The outcomes furnish baseline knowledge concerning bacterial profiles in poultry refrigeration environments in Al-Baha, bearing potential repercussions for food safety strategies in analogous contexts.

## 2. Materials and Methods

### 2.1. Samples Collection

A total of 150 swab samples were collected from 150 supermarket from the internal surfaces of refrigerators specifically designated for poultry and chicken storage in supermarkets located in Al-Mandaq city, within the Al-Baha region of Saudi Arabia. Sample collection was carried out during 72 h (2–4 May 2024), using sterile cotton swabs pre-moistened with sterile normal saline to ensure effective recovery of surface-associated microorganisms as documented by Rose et al. [29]. Swabbing was systematically conducted across various internal areas of the refrigeration units, with particular attention given to surfaces that were in direct contact with poultry products or frequently exposed to potential contamination, including corners, shelves, and inner walls. This targeted sampling approach was designed to maximize the detection of microbial presence in zones most likely to harbor foodborne pathogens and spoilage organisms, thereby providing a representative assessment of hygiene conditions within poultry retail refrigeration environments. Following collection, all swab samples were immediately transferred into sterile transport tubes and placed in insulated containers maintained at 4 °C using ice packs to preserve microbial viability [30]. Samples were transported under these controlled conditions to the microbiology laboratory within two hours of collection, this aligns with the guidelines outlined in the FDA Bacteriological Analytical Manual, which are designed to avert temperature-induced artifactual alterations in microbial populations. This prompt and temperature-controlled transport protocol was implemented to minimize potential changes in microbial composition and ensure the accurate recovery and analysis of viable microorganisms present on the sampled surfaces.

### 2.2. Primary Culture and Bacterial Enrichment

Upon arrival at the laboratory, each swab sample was immediately inoculated into sterile Nutrient Broth (Oxoid, UK) and incubated at 37 °C for 24 h. This enrichment step was performed to facilitate the recovery and proliferation of a wide spectrum of bacterial species, including those present in low numbers or in a stressed physiological state. The use of a non-selective enrichment medium such as Nutrient Broth provides favorable growth conditions, thereby enhancing the likelihood of detecting diverse microbial populations during subsequent isolation and identification procedures.

### 2.3. Selective and Differential Media Culturing

Following enrichment, aliquots from the incubated broths were subcultured onto selective and differential agar media to facilitate the isolation and presumptive identification of pathogenic bacteria. Eosin Methylene Blue (EMB) Agar was used for the differentiation of Escherichia coli and other lactose-fermenting Enterobacteriaceae, based on characteristic colony morphology and metallic sheen. Salmonella-Shigella (SS) Agar was employed for the selective isolation of *Salmonella* and *Shigella* species, utilizing its inhibitory components to suppress competing flora. HiChrom Staphylococcus Agar was utilized to selectively isolate Staphylococcus species, enabling chromogenic differentiation based on colony pigmentation. All inoculated plates were incubated aerobically at 37 °C for 24 h, after which colony morphology was examined and recorded to support presumptive identification prior to further biochemical and molecular analyses.

### 2.4. Bacterial Identification

A total of 29 representative bacterial isolates exhibiting distinct morphological characteristics on the selective and differential media were carefully selected for comprehensive species-level identification. All 29 isolates were subjected to identification using the VITEK^®^ 2 Compact system (bioMérieux, Marcy-l’Étoile, France), an automated platform that performs high-throughput biochemical profiling. The identification process was carried out in accordance with the manufacturer’s instructions. This system enables rapid and accurate classification of bacterial isolates based on their metabolic and enzymatic activity patterns, providing reliable confirmation of presumptive identifications obtained from culture-based methods.

### 2.5. Molecular Characterization

To validate and confirm the VITEK^®^ 2 species-level identifications and enable detailed phylogenetic analysis, all 29 bacterial isolates were subsequently subjected to 16S rRNA gene sequencing. This dual-approach identification strategy ensures robust and accurate taxonomic assignment of all recovered isolates. After that, genomic DNA was extracted from overnight cultures of bacterial isolates grown in Luria–Bertani (LB) broth. One milliliter of each culture was transferred to a sterile microcentrifuge tube and centrifuged at 10,000 rpm for 5 min at 4 °C. The resulting supernatant was discarded, and the cell pellet was resuspended in 200 µL of TES buffer containing 10 mM Tris-HCl (pH 7.5), 10 mM EDTA (pH 8.0), and 0.5% SDS, using gentle pipetting to ensure complete homogenization. To facilitate cell wall lysis, 20 µL of lysozyme solution (10 mg/mL) was added to each tube, followed by gentle mixing and incubation in a water bath at 37 °C for 2 h. Subsequently, 20 µL of proteinase K solution (10 mg/mL) was added, and the mixture was incubated for an additional 2 h at 37 °C, followed by cooling for 5 min at room temperature. To precipitate cellular proteins and other contaminants, 250 µL of sodium acetate solution was added to each tube, followed by centrifugation at 8000 rpm for 5 min at 4 °C. The clear supernatant was carefully transferred to a new sterile tube. An equal volume of chloroform/isoamyl alcohol (24:1) was then added, and the mixture was gently inverted several times for thorough mixing. After centrifugation at 8000 rpm for 5 min at 4 °C, the upper aqueous phase was transferred to a fresh tube. DNA was precipitated by adding an equal volume of isopropanol and storing the mixture overnight at −20 °C. DNA pellets were recovered by centrifugation at 10,000 rpm for 5 min, then air-dried at room temperature for approximately 60 min. Finally, the dried DNA pellet was resuspended in 50 µL of nuclease-free water and stored at −20 °C until further use. Then the 16S rRNA gene was amplified using universal primers 27F and 1492R via polymerase chain reaction (PCR). Amplified products were subsequently purified and subjected to Sanger sequencing to confirm bacterial identity and explore phylogenetic relationships. Resulting sequences were analyzed using the BLAST (version 2.15.0) (Basic Local Alignment Search Tool) algorithm against the NCBI nucleotide database to achieve accurate taxonomic classification based on sequence homology.

### 2.6. Inter Simple Sequence Repeat (ISSR) Analysis

Primer selection was based on preliminary screening of multiple ISSR primers from published literature. Several universal primers were initially tested on a representative subset of isolates, and only those primers that produced clear, reproducible, and highly polymorphic banding patterns were retained for full analysis. The final panel of six ISSR primers (HB13, SAS1, TERRY, UBC827, UBC809, and UBC811) represents those that demonstrated the most promising and consistent results for distinguishing among the bacterial isolates, as listed in (Table 1), to assess the genetic fingerprinting of the bacterial isolates under investigation. PCR amplification was performed in a 25 µL reaction volume containing 1× PCR buffer, 1.5 mM MgCl2, 2 mM dNTPs, 1 U Taq DNA polymerase, 25 ng of template DNA, and 1 µM of each ISSR primer. The thermal cycling protocol began with an initial denaturation at 94 °C for 5 min, followed by 36 cycles consisting of denaturation at 94 °C for 60 s, annealing at 44 °C for 60 s, and extension at 72 °C for 1.5 min. A final extension step was carried out at 72 °C for 10 min to ensure complete amplification of all fragments. The resulting PCR products were separated by electrophoresis on a 1.5% agarose gel containing 0.5 µg/mL ethidium bromide in 1× TBE buffer. Electrophoresis was conducted at 90 V, and DNA banding patterns were visualized under UV light and documented using a gel documentation system. Molecular fingerprinting analysis and band scoring was performed using TotalLab TL 120 software, which applied the UPGMA algorithm based on the Jaccard similarity coefficient to generate dendrograms and illustrate genetic relationships among the isolates.

### 2.7. Random Amplified Polymorphic DNA (RAPD) Analysis

Primer selection was conducted through systematic preliminary evaluation of multiple RAPD decamer primers from established primer sets (OPA, OPW, and OPU series). Initially, numerous primers were tested on a representative subset of diverse bacterial isolates. Only those primers that produced clear, reproducible, and highly polymorphic banding patterns with minimal background noise or smearing were selected for the complete analysis. The final panel of six RAPD primers (OPA-08, OPA-10, OPA-18, OPW-08, OPW-11, and OPU-15) represents those that yielded the most promising and consistent results, as detailed in (Table 2). PCR amplification was carried out in a 25 µL reaction mixture containing 1× PCR buffer, 1.5 mM MgCl2, 2 mM dNTPs, 1 U Taq DNA polymerase, 25 ng of template DNA, and 1 µM of each primer. The PCR cycling conditions consisted of an initial denaturation at 94 °C for 5 min, followed by 36 cycles of denaturation at 94 °C for 60 s, annealing at 32 °C for 60 s, and extension at 72 °C for 1.5 min. A final extension step was performed at 72 °C for 10 min to ensure complete amplification. PCR products were separated by electrophoresis on a 1.5% agarose gel containing 0.5 µg/mL ethidium bromide in 1× TBE buffer. Electrophoresis was conducted at 90 V, and DNA bands were visualized and documented under UV illumination using a gel documentation system. Molecular fingerprinting analysis and band scoring was performed using TotalLab TL 120 software, which applied the UPGMA algorithm based on the Jaccard similarity coefficient to generate dendrograms and illustrate genetic relationships among the isolates.

## 3. Results

### 3.1. Bacterial Contamination of Poultry Refrigeration

Al-Baha region of Saudi Arabia is distinguished by its mountainous topography and significant seasonal temperature variations, both of which may complicate the preservation of cold chain integrity within retail food systems. Such environmental conditions can potentially affect bacterial persistence and proliferation during the processes of food storage and distribution [31]. The analysis of microbial isolates derived from poultry refrigeration units within the region (Table 3) demonstrated a predominance of the *Salmonella* enterica complex, representing 51.72% of all identified bacterial strains. This observation is consistent with international concerns about the involvement of *Salmonella* in poultry contamination and its consequent public health implications. Among the serovars isolated, *Salmonella* Enteritidis (isolates B2, B3, B5, B6, B7, B8, B13, B17) was the most frequently observed. This serovar is distinguished by its capacity to endure refrigeration temperatures, with established survival at 4 °C and minimal inhibition under cold storage conditions. Additionally identified was *Salmonella typhimurium* (isolate B24), a well-documented etiological agent of gastroenteritis. Other serovars included *Salmonella waycross* (B1, B9, B11), frequently noted in regional distributions; *Salmonella minnesota* (B4, B38), commonly associated with specific poultry sources and processing environments; and *Salmonella dublin* (B34), which, though more often associated with cattle, occasionally appears in poultry production systems.

In addition to *Salmonella*, isolates affiliated with the *Escherichia* complex represented 20.7% of the identified bacteria, suggesting potential fecal contamination and inadequate hygiene during poultry handling and storage. *E. coli* (B12, B14, B19, B36), a recognized indicator organism, acts as a marker for fecal pollution and may be found concurrently with other enteric pathogens. Moreover, the presence of *Escherichia fergusonii* (B18) and *Escherichia* sp. (B10) underscores emerging public health threats. Significantly, *Bacillus* species (B16, B30, B33) were detected, encompassing spore-forming organisms capable of enduring extreme environmental conditions. Their ability to form resilient spores allows persistence even during extended refrigeration periods. *Citrobacter* species (B21, B25), part of the Enterobacteriaceae family, were also identified. These bacteria are frequently associated with contaminated water and unsanitary handling practices, and they are known for their ability to acquire resistance in food-processing environments. Additional isolates such as *Arthrobacter* sp. (B20), a soil-associated organism, suggest pathways for environmental contamination. Furthermore, *Enterobacter* cloacae (B15) and *Enterobacter* sp. (B31), typically linked with nosocomial infections, pose additional concerns due to their inherent resistance to multiple antibiotics.

**Figure 1:**
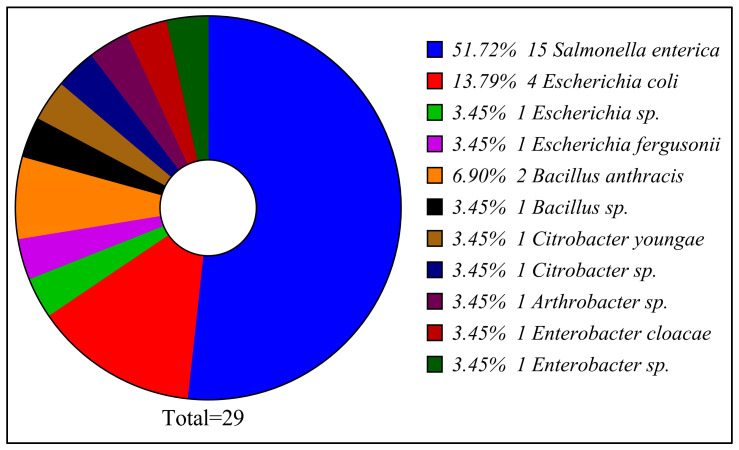
Taxonomic composition of the 29 bacterial isolates obtained from poultry refrigeration units in the Al-Baha region.

The pictogram depicted in (Figure 1) illustrates the taxonomic distribution of the identified isolates, with a pronounced dominance of *Salmonella enterica* (51.72%). This substantial prevalence reflects the study’s primary focus and emphasizes the public health significance of *Salmonella* in poultry-associated contamination. Additionally, *E. coli* accounts for 13.79% of the isolates, frequently co-occurring with *Salmonella* in both clinical and environmental contexts. Two additional *Escherichia* taxa, *E. fergusonii* and *Escherichia* sp., each contribute 3.45%, together forming 6.9% of the dataset, indicating a limited presence of non-coli *Escherichia* species. *Bacillus* species, including *Bacillus anthracis* (6.90%) and *Bacillus* sp. (3.45%), collectively comprise 10.35% of isolates. Their presence highlights the occasional occurrence of Gram-positive bacteria and the resilience of spore-forming organisms in refrigerated environments. Low-level detection of *Citrobacter youngae* (3.45%) and *Citrobacter* sp. (3.45%) indicates a minor representation within the Enterobacteriaceae family, while isolates of *Arthrobacter* sp., *Enterobacter cloacae*, and *Enterobacter* sp. (each 3.45%) reflect the occasional occurrence of environmental or opportunistic pathogens.

### 3.2. ISSR Investigation Between Isolates

#### Primer-Specific Amplification and Polymorphism Profiles

**Figure 2:**
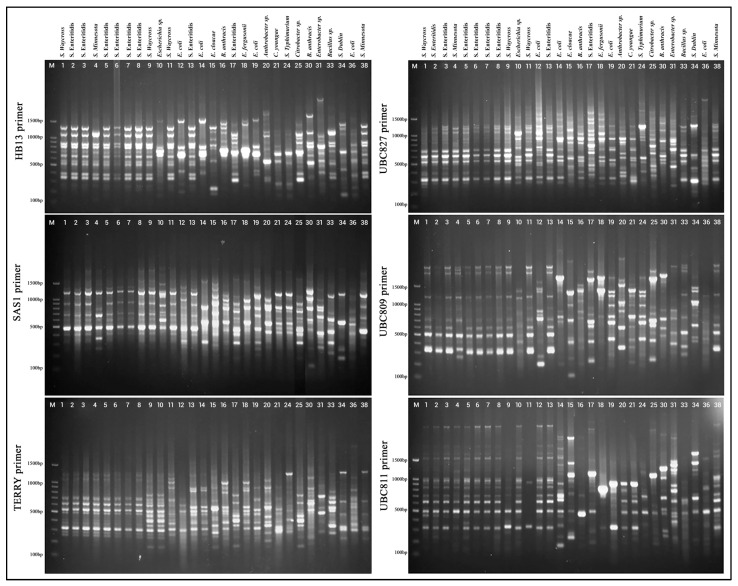
ISSR-PCR gel electrophoresis showing banding patterns of bacterial isolates from poultry refrigeration units.

The genetic diversity of the bacterial isolates was evaluated at the molecular level utilizing ISSR primers, as illustrated in (Figure 2), with each primer demonstrating varying degrees of amplification efficiency, banding resolution, and discriminatory capacity. The primers are categorized into two groups based on the presence of one or two additional bases at the 3′ end, referred to as anchors, which enhance specificity. The first group encompasses the HB13 Primer, which consistently produced robust amplification across the majority of isolates, generating multiple well-defined bands ranging from 200 bp to over 1500 bp. Notably, it exhibited high polymorphism, resulting in distinct banding patterns among most isolates, especially within the mid-to-high molecular weight range (500–1500 bp). Its high discriminatory power was evident in isolates such as 12 (*E. coli*), 13 and 17 (*Salmonella* Enteritidis), 15 (*Enterobacter cloacae*), 16 and 30 (*Bacillus anthracis*), 18 (*E. fergusonii*), 19 and 36 (*E. coli*), 20 (*Arthrobacter species*), 21 (*Cronobacter youngae*), 24 (*Salmonella typhimurium*), 25 (*Citrobacter species*), 31 (*Enterobacter species*), 33 (*Bacillus* species), 34 (*Salmonella dublin*), and 38 (*Salmonella minnesota*), which displayed unique and diverse profiles indicative of substantial genetic variation. SAS1 Primer, on the other hand, demonstrated similarly high amplification quality and polymorphism, effectively resolving genetic differences among isolates. Fragment sizes ranged from 200 bp to 1500 bp with clear and well-separated bands. Isolates 12–21, 24–25, and 30–38 displayed considerable banding variation, further supporting its robustness for discriminating genetically distinct strains. The primer performed comparably to HB13 in both band resolution and diversity. Amplification with the TERRY primer was moderate to good, although some isolates exhibited fainter or fewer bands compared to HB13 or SAS1. Polymorphism was moderate, with several isolates displaying overlapping patterns. Band sizes were typically within the 200–1500 bp range, but occasional smearing was observed. Notably, isolates 1–11 (comprising various Salmonella and Escherichia strains) shared relatively similar profiles, indicating reduced resolving power among closely related taxa. However, isolates 12 and above displayed greater diversity.

Second group designated as UBC827 Primer, this primer generated highly polymorphic and informative banding patterns across all isolates, exhibiting strong, discrete bands mostly within the 200–1500 bp range. UBC827 showed particular efficacy in distinguishing isolates that seemed analogous with the TERRY primer, highlighting its significance in revealing concealed genomic variation. Its great resolution and substantial information richness designate it as one of the most effective primers in this research. While UBC809 produced many robust bands spanning a broad size range (200 bp to over 1500 bp), with good amplification efficiency and polymorphism. It repeatedly demonstrated distinct genetic profiles for various isolates, providing exceptional discrimination capability. In conjunction with UBC827, it is ranked among the most polymorphic and informative primers used in this investigation. UBC811, similar to UBC809 and UBC827, UBC811 exhibited substantial amplification and significant polymorphism, with pronounced bands located between the 200–1500 bp range. It offered significant distinction among isolates, hence affirming its effectiveness in evaluating intra- and interspecies genetic diversity. The patterns exhibited well defined genetic profiles, highlighting their significance in molecular fingerprinting.

**Figure 3:**
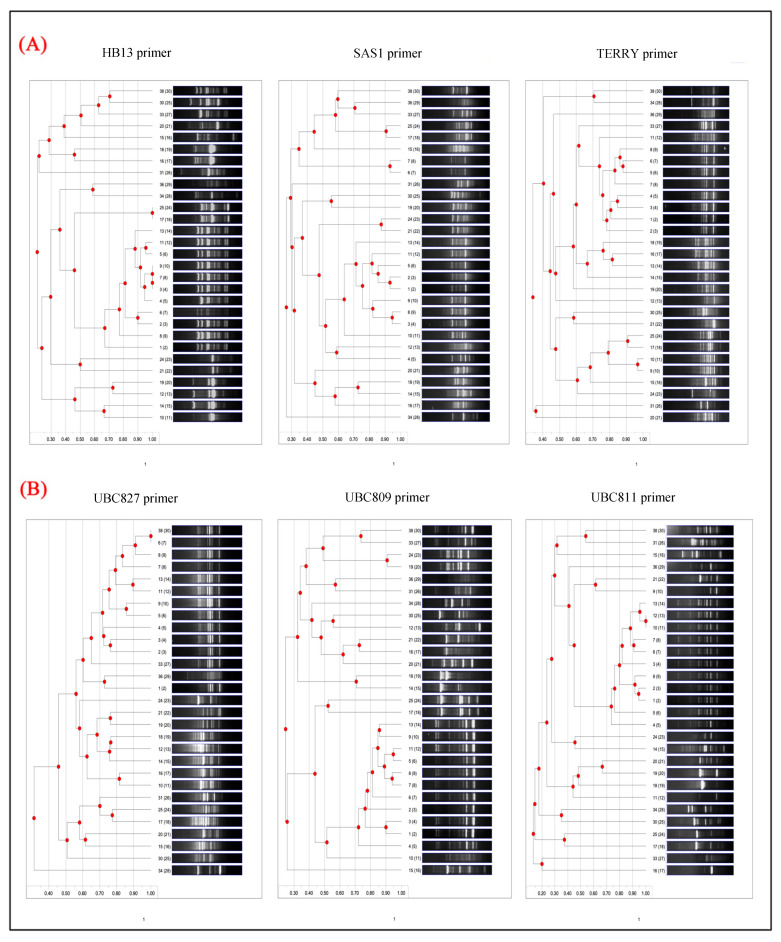
The ISSR dendrograms generated from the banding patterns of each primer reveal the genetic relationships among the 29 bacterial isolates. Panel (**A**) illustrates commonly utilized customized primers, while panel (**B**) presents standardized primers derived from the University of British Columbia (UBC) primer set.

The primer-specific dendrogram analysis (Figure 3), The dendrograms derived from the banding patterns of each primer elucidate the genetic relationships among the 29 bacterial isolates. These hierarchical structures are indicative of the polymorphism and discriminatory capacity observed in the gel electrophoresis profiles, revealing clustering patterns that align with species diversity and intra-species variation. The dendrogram generated from HB13 amplification displays several well-defined clusters, suggesting considerable genetic diversity among the isolates. This primer exhibits high discriminatory power, effectively distinguishing even closely related strains into distinct sub-clusters that displayed on the dendrogram nodes at the points of branch divergence, with values ≥75% considered as indicating strong cluster support, values of 60–74% indicating moderate support, and values <60% indicating weak or unsupported clusters. Isolates with gel profiles that are visually similar tend to cluster in proximity, whereas isolates with highly polymorphic patterns form branches that are more distantly related. For example, isolates 36 (*E. coli*) and 38 (*S. Minnesota*) are clearly delineated into separate branches, underscoring their genetic divergence as and their values is <60%. The dendrogram can be depicted as comprising four principal clusters. The central cluster predominantly contains the *Salmonella* genera, the cluster directly above it includes a mixture of *Bacillus* and *Arthrobacter*, and the cluster at the bottom is primarily composed of *E. coli*. While the clustering pattern of the SAS1 dendrogram mirrors that of HB13, it additionally reveals considerable genetic diversity and the presence of multiple distinct clusters. Its strong discriminatory capability is demonstrated in its effectiveness at differentiating closely related isolates. Certain *S.* Enteritidis isolates from tightly knit clusters, whereas others are dispersed across separate branches, indicating intra-species genetic variability. The dendrogram substantiates the substantial polymorphism observed in the SAS1 gel profiles. In contrast, the TERRY dendrogram displays more compact clustering, particularly at higher similarity coefficients, suggesting a lower overall resolution and a diminished ability to detect fine-scale genetic differences. The moderate discriminatory power aligns with the gel analysis, wherein TERRY exhibited fewer and occasionally weaker bands. Larger, less resolved clusters are apparent, indicating that this primer may be more appropriate for identifying broader phylogenetic relationships rather than nuanced strain-level distinctions.

The dendrogram corresponding to UBC827 is characterized by a highly resolved structure, featuring numerous branches and distinctly separated sub-clusters. This primer exhibits exceptional discriminatory capabilities, effectively distinguishing nearly all isolates. Even strains that appeared similar when analyzed with other primers are distinctly separated in this instance. The resulting dendrogram offers a comprehensive genetic fingerprint for each isolate, substantiating its utility in precise strain differentiation and phylogenetic mapping. Analogous to UBC827, the dendrogram associated with UBC809 demonstrates excellent resolution, with a clear demarcation of isolates into multiple distinct groups. Its considerable discriminatory power is evident through the richness of the tree structure and the extensive diversity it encapsulates. These clustering patterns further validate the primer’s high polymorphism and its efficacy in discriminating among bacterial taxa. The dendrogram for UBC811 presents a well-structured and distinctly resolved tree, comparable to those generated by UBC809 and UBC827. It demonstrates a high discriminatory ability, accurately distinguishing between isolates and highlighting both inter- and intra-species variation. The consistent separation of strains into distinct clusters affirms the significant informative value of this primer for molecular typing and diversity analysis.

**Figure 4:**
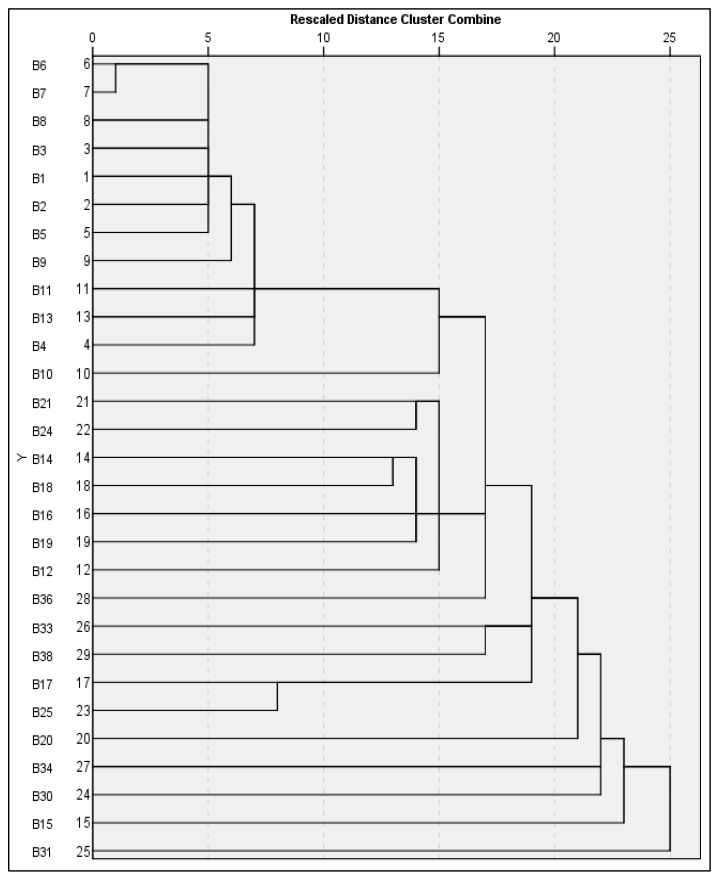
Illustrates the dendrogram resulting from the clustering of variables derived from 29 bacterial samples, utilizing all ISSR primers. The cluster analysis of these variables is conducted based on their degree of similarity.

The single linkage dendrogram derived from ISSR marker profiles (Figure 4) demonstrates a distinct stratification of genetic relationships among the bacterial isolates. The initial major cluster, formed at a distance threshold of ≤5, encompasses isolates B1 (*S. enterica serovar Waycross*), B2, B3, B5, B6, B7, and B8 (both *S. enterica serovar* Enteritidis). This cluster represents the most genetically homogeneous entity within the dendrogram. The minimal polymorphism observed among these isolates aligns with the clonal nature of *S.* Enteritidis, a serovar characterized by its limited genetic variability. Their proximal clustering implies a substantial degree of genomic conservation and potentially recent common ancestry; additionally, a second cluster contain isolates B9 and B11, representing (*S. waycross*), as well as B13 (*S. enteritidis*), B4 (*S. minnesota*), and B10 (*Escherichia* sp.), are included at a more genetically diverse group forms within the 10–15 distance range. The inclusion of multiple Salmonella serovars alongside *Escherichia* highlights broader diversity within the Enterobacteriaceae family and marks a transition from clonal to more heterogeneous genomic structures. These relationships suggest evolutionary divergence within and between these genera. At greater genetic distances (above 15), the fourth major grouping encompasses the most divergent strains in the dataset. This group includes B21 (*Citrobacter youngae*), B24 (*S. typhimurium*), B14, B18, and B19 (various *Escherichia* species), B16 (*Bacillus anthracis*), and B20 (*Arthrobacter* sp.). These isolates are positioned on separate branches of the dendrogram, reflecting significant taxonomic and genetic differences. The separation of *Bacillus* and *Arthrobacter* both Gram-positive genera from Gram-negative Enterobacteriaceae members further supports the robustness of ISSR markers in resolving broad phylogenetic relationships. Significantly, the dendrogram demonstrates robust taxonomic concordance, wherein Salmonella enterica serovars exhibit a propensity to cluster together. The close assembly of *S.* Enteritidis isolates at low distance thresholds corroborates their clonal behavior, whereas *S. typhimurium* (B24) is positioned on a separate branch, indicative of notable serovar-level divergence. Notably, *S. dublin* (B34) and *S. minnesota* (B38, B4) occupy intermediate positions, implying shared ancestral traits yet exhibiting differentiated genomic profiles. This serovar-specific clustering mirrors established phylogenetic relationships within Salmonella. For example, *S.* Enteritidis isolates (B1, B2, B5-B8, B13, B17) are consistently located within compact, low-distance clusters, reinforcing their genetic homogeneity. Conversely, the separation of *S. typhimurium* and *S. dublin* underscores their evolutionary divergence from *S.* Enteritidis. Similarly, *S. minnesota* isolates are found in more dispersed positions, suggesting intermediate levels of divergence within the genus.

### 3.3. RAPD Investigation Between Isolates

#### 3.3.1. RAPD-PCR Gel Electrophoresis Analysis

**Figure 5:**
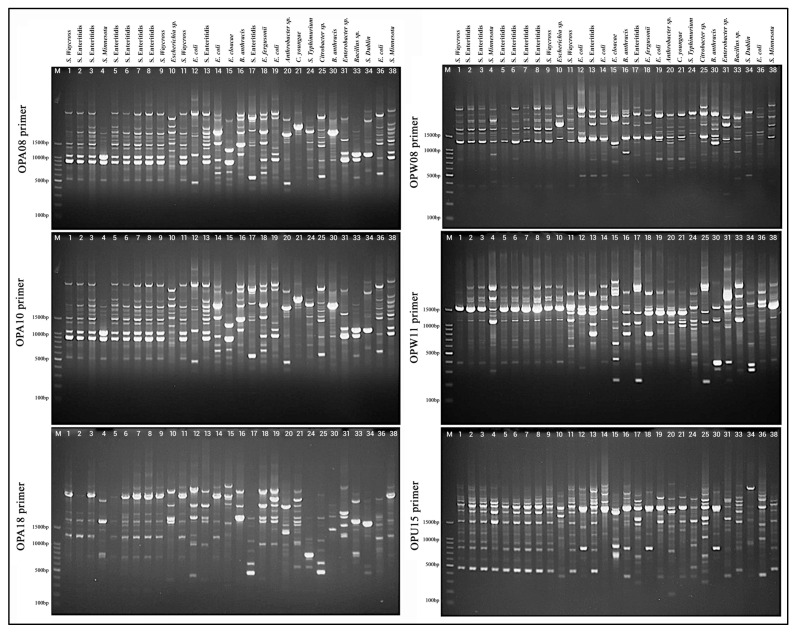
Photograph of the gel electrophoresis of RAPD products for all isolates derived from poultry refrigeration units.

Each primer demonstrated differential capabilities in distinguishing bacterial strains based on the quantity, intensity, and distribution of DNA fragments (Figure 5). The OPA08 primer yielded a diverse array of bands across the bacterial samples. Strains located in lanes 1–9, representing *S. waycross*, *S. enteritidis*, and *S. minnesota*, exhibited relatively similar banding profiles, suggesting genetic relatedness. In contrast, *E. coli* strains (lanes 12–13) and *Bacillus anthracis* (lane 16) exhibited unique and distinct profiles, indicating substantial genetic divergence. Polymorphic banding patterns were again observed with OPA10. Although *S. enteritidis* isolates (lanes 4–9) shared several common bands, minor differences were evident, underscoring intra-species diversity. Other species, such as *Enterobacter* cloacae (lane 15) and *Citrobacter* sp. (lane 24), exhibited markedly different profiles compared to the *Salmonella* strains, emphasizing inter-genus variability. The OPA18 primer offered enhanced resolution of genetic relationships. Whilst *S. enteritidisstrains* retained some conserved bands, variation persisted, illustrating subtle genomic differences. Notably, samples from lanes 30–38, including *Bacillus* sp., *S. Dublin, E. coli*, and *S. Minnesota*, generated distinct and often simpler banding patterns, suggesting significant genetic divergence from other groups. OPW08 produced clear and reproducible bands across most samples. *Salmonella* strains, including *S. Waycross, S. enteritidis,* and *S. Minnesota*, maintained comparable profiles with sufficient variation to permit discrimination between isolates. Meanwhile, non-Salmonella bacteria showed distinct profiles, further corroborating RAPD’s efficacy in differentiating across genera. Employing OPW11, robust amplification profiles were observed. While *S.* Enteritidis isolates displayed a characteristic set of bands with minor variations, *E. coli* and other Gram-negative bacteria such as *Enterobacter* and *Citrobacter* species demonstrated clearly distinct profiles, sometimes featuring high-molecular-weight bands or an absence of Salmonella-associated bands. The final primer, OPU15, generated comparatively fewer bands for several strains, particularly among *Salmonella* isolates. Nevertheless, the primer is effectively distinguished between bacterial genera. For example, *Bacillus* sp. (lane 30) and *Arthrobacter* sp. (lane 20) exhibited distinctly simpler banding profiles compared to the complex patterns observed in Enterobacteriaceae, contributing to the overall discriminatory capacity.

#### 3.3.2. RAPD-Based Dendrogram Analysis

**Figure 6:**
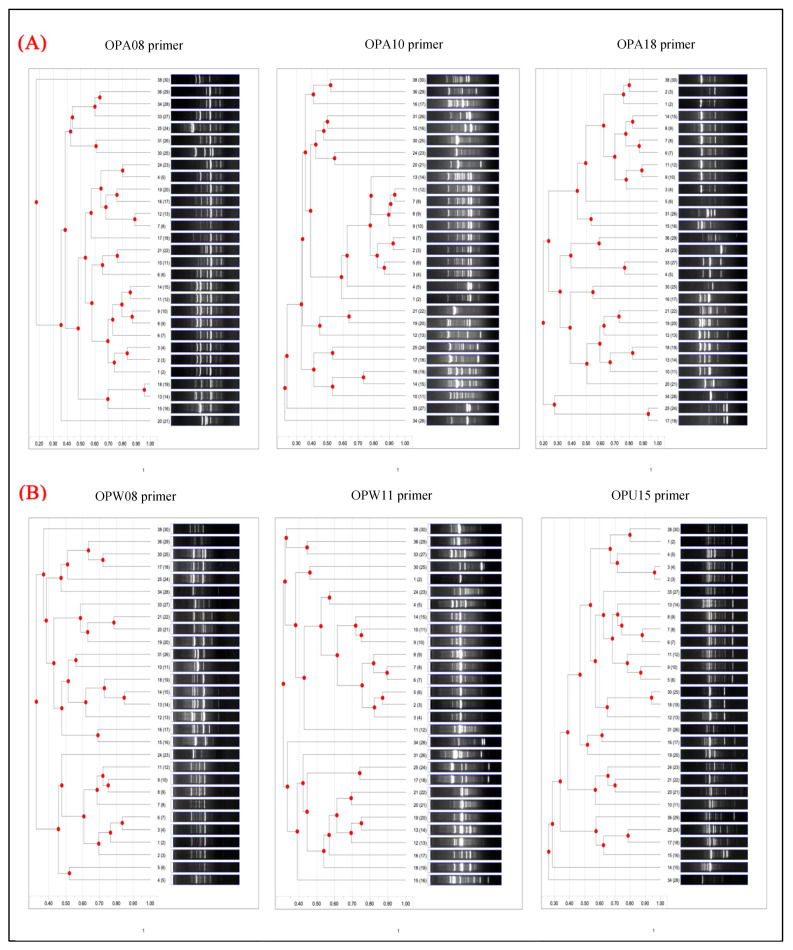
The dendrograms derived from the RAPD banding patterns of each primer illustrate the genetic relationships among the 29 bacterial isolates. Panel (**A**) depicts the OPA group characterized by a 70% purine content, whereas panel (**B**) displays other standardized primers with a purine content ranging from 50% to 60%.

The dendrogram analysis derived from RAPD profiles (Figure 6) delineates distinct clustering patterns and genetic affiliations among the tested bacterial isolates. This comparative assessment, structured around the OPA, OPW, and OPU primer series, uncovers varying degrees of genetic resolution, ranging from genus-level aggregation to fine-scale strain differentiation. Panel A: OPA Series Primers, the dendrogram produced with primer OPA08 exhibits a complex hierarchical configuration, forming multiple well-separated genetic clusters. The similarity coefficients extend over a broad spectrum (0.45–0.95), signifying both closely related and genetically divergent strains. Remarkably, 4–5 distinct groups emerge, some of which contain singleton clusters that likely signify genetically unique isolates. This primer demonstrates high discriminatory capacity, particularly in distinguishing between bacterial genera. Conversely, the OPA10 primer generates a more equilibrated dendrogram with distinct intermediate-level clustering. The genetic distance is distributed incrementally from 0.50 to 0.90, and 3–4 major clades are consistently delineated. This primer effectively captures both strain-level differentiation and broader taxonomic relationships, evidencing a strong phylogenetic signal. The OPA18 primer dendrogram portrays tighter clustering, with a predominant large cluster encompassing the majority of Salmonella enterica isolates. A bimodal similarity pattern is observed: while most strains exhibit high similarity (>0.80), several isolates are positioned at the margins with significant divergence. This primer provides excellent resolution for identifying taxonomic outliers but exhibits somewhat restricted within-group discrimination.

While Panel B: OPW/OPU Series Primers The dendrogram derived from OPW08 demonstrates extensive genetic diversity among isolates, characterized by dispersed clustering across the similarity spectrum. Comprising 5–6 distinct groups alongside numerous intermediate branches, the tree structure signifies a gradual evolutionary divergence. This primer is particularly beneficial for inter-genus differentiation owing to its robust resolution and representation of genetic heterogeneity. OPW11 generates a dendrogram of moderate complexity, exhibiting several well-defined genetic clusters. The similarity range (0.50–0.85) indicates balanced diversity. The resulting clusters are internally consistent, providing valuable insights into both inter- and intra-species genetic relationships, rendering OPW11 apt for epidemiological typing purposes. Conversely, OPU15 exhibits a more fragmented clustering pattern, characterized by multiple small groups as opposed to dominant clusters. The extensive similarity span (0.40–0.90) reflects substantial genetic variability among the isolates. Its dendrogram structure facilitates strain-level differentiation and offers exceptional resolution for identifying subspecies-level variations, rendering it an especially potent primer for fine-scale phylogenetic studies.

Cross-primer analysis reveals that each RAPD primer elucidates distinct facets of bacterial diversity. Such as conservative primers such as OPA18 primarily resolve major taxonomic divisions, whereas primers like OPA10 and OPW11 provide balanced discrimination across hierarchical levels. Conversely, highly discriminatory primers such as OPW08 and OPU15 capture intricate genetic differences, including strain-specific polymorphisms. The distribution of similarity coefficients across all dendrograms provides additional insight. High similarity values (>0.80) account for 25–30% of strain pairs, predominantly consisting of *Salmonella enterica* Enteritidis isolates. Moderate similarity (0.60–0.80) constitutes nearly half of all relationships, highlighting genetically related yet distinct strains. Low similarity (<0.60) is observed in 20–25% of comparisons, indicating significant divergence and supporting the existence of taxonomically distant organisms. The dendrogram outcomes broadly align with established bacterial taxonomy. At the genus level, Salmonella enterica isolates cluster together across all primers, with *Escherichia* species forming intermediate groups. In contrast, non-Enterobacteriaceae members such as Bacillus and Arthrobacter appear as outliers, which is consistent with their evolutionary distance. Within the Salmonella genus, serovar-specific patterns are evident. *S. enterica* Enteritidis demonstrates high genetic homogeneity (similarity >0.75) across multiple primers, while *S. enterica* Typhimurium consistently forms a distinct cluster, showcasing clear serovar-level discrimination. These findings coincide with known Salmonella phylogenies and endorse the utility of RAPD as an instrument for both genotypic and phylogenetic assessment.

**Figure 7:**
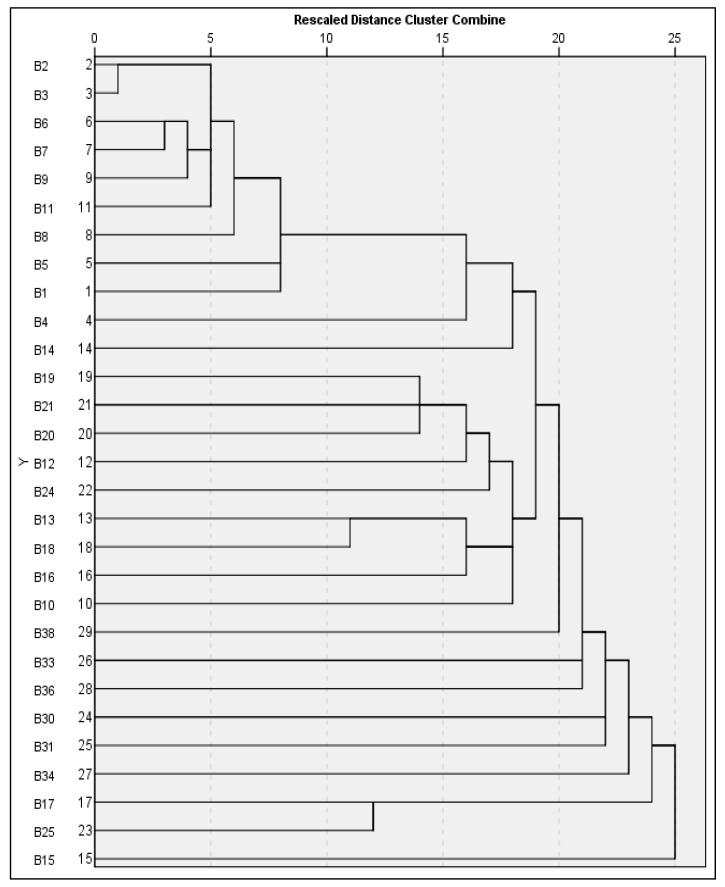
Presents a dendrogram illustrating the clustering of variables from 29 bacterial samples, derived from the analysis of all RAPD primers. The cluster analysis of these variables is conducted based on their similarities.

The hierarchical clustering of bacterial isolates, based on the aggregated RAPD data (Figure 7), elucidated distinct genetic assemblages, each indicative of varying levels of taxonomic similarity and evolutionary divergence. At the ultra-high similarity threshold (distance 0–5), a primary cluster was discerned, comprising isolates B2 and B3. Both strains are classified as *Salmonella enterica* subsp. enterica serovar Enteritidis and exhibit similarity coefficients exceeding 95%. This close proximity in clustering suggests they are likely clonal isolates originating from a common epidemiological source, corroborated by their nearly identical RAPD profiles across all six primers, signifying minimal polymorphism and a recent common ancestry. Extending into the high similarity range (distance 5–10), a secondary cluster emerged, incorporating isolates B6, B7, B9, B11, and B8. These strains represent a mix of *Salmonella serovars*, predominantly Enteritidis and Waycross, and constituted a coherent group. The clustering pattern signifies the presence of shared genetic elements, while still facilitating adequate resolution to distinguish individual strains. This implies they belong to related but genetically discernible lineages within the *S. enterica* species complex, providing valuable insights into intra-species evolutionary dynamics. In the moderate similarity range (distance 10–15), an intermediate cluster composed of B5, B1, B4, and B14 was observed. These strains include both *Salmonella* (Enteritidis and Minnesota serovars) and *E. coli*, representing members of the Enterobacteriaceae family. This grouping appears to bridge closely related *Salmonella* isolates and *Escherichia* species, demonstrating phylogenetic conservation at the family level while maintaining genus-specific divergence. The observed genetic distances reflect moderate evolutionary divergence and indicate shared ancestral traits across these Enterobacteriaceae taxa. As similarity decreases further (distance 15–20), a divergent cluster appears, consisting of B19, B21, B20, B12, and B24. This group displays significant taxonomic heterogeneity, including isolates of *Escherichia*, *Citrobacter*, *Arthrobacter*, and *Salmonella Typhimurium*. The broad genetic divergence within this group, evidenced by lower similarity coefficients, reflects distant phylogenetic relationships. The high discriminatory power of this clustering supports its application in epidemiological investigations and confirms the genetic distinctiveness of these strains. At the highest level of diversity (distance >20), a set of highly divergent strains forms a maximum diversity cluster, composed of 13 isolates. These include members of *Bacillus*, *Enterobacter*, and other diverse Enterobacteriaceae, and are characterized by extended branching and multiple singleton positions in the dendrogram. This pattern illustrates distant evolutionary relationships and highlights the ability of the RAPD approach to achieve maximal strain-level resolution across wide taxonomic distances.

Overall, the ISSR and RAPD primer sets showed high levels of polymorphism, with differences seen between the ISSR and RAPD sets as well as among each set individually as summarized in (Table 4). The ISSR primers generally yielded more bands than the RAPD primers. UBC827 produced the largest number of bands (22), and also the highest level of polymorphism (90.9%) followed by UBC809 (89.5%), SAS1 (88.9%), and UBC811 (88.2%) which indicated that they could be used to differentiate very closely related isolates. In contrast, TERRY was the primer producing the fewest number of bands (12) and the lowest percentage of polymorphic bands (66.7%), and therefore it is the primer with the least ability to identify variations. When comparing the primers in the RAPD system, OPW-08 produced the highest percentage of polymorphism (85.0%), followed by OPU-15 (84.6%), and then OPA-10 (83.3%), which demonstrated that those three primers would have been the best to use when looking for genetic differences. OPA-18 produced the lowest percentage of polymorphism (71.4%) demonstrating less resolving ability. This higher percentage of polymorphism from many of the primers shows that both ISSR and RAPD are useful for detecting genetic variation among the isolates. Additionally, the integration of the ISSR and RAPD systems to detect genetic variation provides a better understanding of the strain-level heterogeneity present in the bacteria.

**Figure 8:**
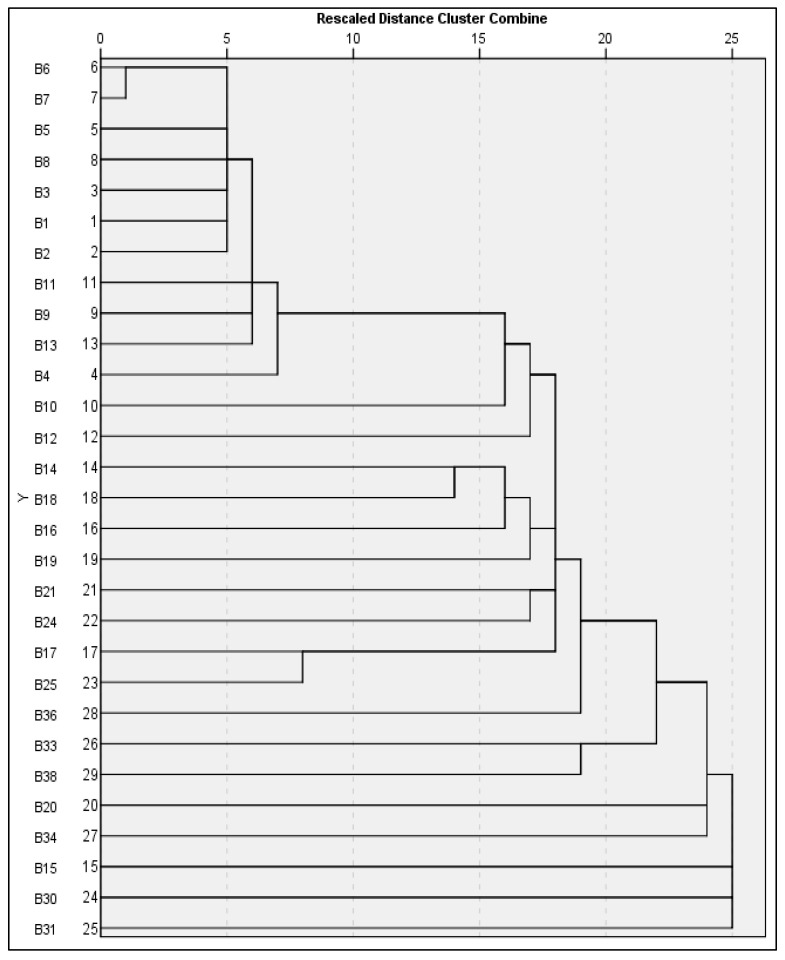
Dendrogram illustrates the clustering of variables derived from 29 bacterial samples, utilizing all ISSR and RAPD primers. The cluster analysis of these variables is performed based on their degree of similarity.

Use of ISSR and RAPD markers together (Figure 8) offers a comprehensive molecular typing tool for identifying bacterial isolates and a better level of discrimination due to increased resolution. For genetic similarities at the most extreme levels (genetic distance = 0–2.5), Isolates B6 and B7 form a first clonal grouping. Both are classified as *Salmonella enterica* subsp., and both have a similarity of greater than 97% when evaluated using the combined markers. Minimal variation was found in both ISSR and RAPD data sets, which suggests a clonal source for the two isolates; possible explanations include a common source of infection, or recent transfer of infections. High levels of genetic uniformity among *S.* Enteritidis populations have been documented previously and supports the fact that *S.* Enteritidis populations are clonally structured. When extending the initial cluster of isolates B5, it shows extremely high levels of genetic similarity (distance 2.5–5) to support placement in the larger Enteritidis group. The similarity among isolates in this cluster further supports the robustness of the dual-marker approach used in this study. ISSR and RAPD allowed discrimination of strains at a similar serovar level while confirming close phylogenetic relationships. A core *Salmonella* cluster, including isolates B8, B3, B1, and B2, are found at the higher genetic similarity range (distance 5–10). These isolates included multiple *Salmonella serovars*, specifically Enteritidis and *Waycross*. Therefore, there are genetic similarities as well as isolate-specific polymorphisms among the isolates in this group. The complementary nature of ISSR and RAPD markers were important for documenting the phylogenetic structure of *Salmonella* relationships and validating previously described *Salmonella* relationships. Isolates B11, B9, B13, and B4 are found at the higher genetic similarity levels (distance 10–15), forming an intermediate *Salmonella* cluster with additional serovars of *Salmonella*, such as *Minnesota* and *Waycross*. Therefore, the evolutionary distances among these isolates suggest divergence within the *Salmonella* complex. As previously stated, the combined use of ISSR and RAPD markers increases the resolution for determining genetic differences and therefore provides evidence of the evolutionary processes occurring among *Salmonella* lineages. At the lower genetic similarity levels (distance 15–20), isolates B10 and B12 (*Escherichia* species) are placed in an intermediate phylogenetic position between *Salmonella* and other members of the Enterobacteriaceae. This finding demonstrates divergence at the genus level, while also illustrating relationships among families. The ISSR-RAPD marker system provides evidence of evolutionary relationships within the Enterobacteriaceae and has sufficient power to discriminate at the genus level. Finally, the high diversity range (distance >20) contains 16 isolates that exhibit extensive branch and/or singleton clusters that represent different genera, such as *Bacillus*, *Enterobacter*, *Citrobacter*, and *Arthrobacter*. The large amount of genetic divergence among these isolates demonstrates the wide phylogenetic breadth provided by the combined ISSR-RAPD analysis and demonstrates the ability of the ISSR-RAPD analysis to discriminate among non-related bacterial taxa. Geographic distribution and serovar composition of isolates from supermarket refrigerated units in Al-Mandaq demonstrate several aspects of the mechanisms of persistence and transmission of pathogens through the retail cold chain. The significant genetic homogeneity of the *S.* Enteritidis clonal complex (isolates B2, B3, B5, B6, B7, B8, B13, B17; 8/29 total isolates; 27.6% of all bacteria; 53.3% of *Salmonella*) all of which are genetically identical (genetic distance = 0–20), likely represents either environmental or transient contaminants with less direct immediate food safety implications than the persistent *S.* Enteritidis lineage.

## 4. Discussion

Zoonotic foodborne illnesses linked to chicken meat represent a significant global concern, posing serious threats to public health and generating considerable economic losses [32]. Among the major bacterial pathogens implicated in these infections are *S.* aureus, *Salmonella* spp., *Campylobacter* spp., *L. monocytogenes*, and *E. coli* [33]. A study by Koesoemo et al. [15] examining microbial contamination in chicken meat sold in local markets in Surabaya found high prevalence rates of *S. aureus* (58.3%), *Salmonella* spp. (48.3%), and *E. coli* (40%). In Ghana, *S. aureus* was detected in 9.2% of raw chicken meat samples, according to Pesewu et al. [34]. Similarly, Kim et al. [35] reported that 47% of chicken meat samples collected in South Korea tested positive for *S. aureus.* Notably, a higher contamination rate was observed in a study by Savariraj et al. [36], where 66.67% of retail chicken meat samples from Chennai, India, were positive for *S. aureus*. The bacterial contamination profile seen in poultry refrigeration units during our investigation in the Al-Baha region of Saudi Arabia reveals significant microbiological difficulties, along with broader trends noted in global research. The predominance of the *Salmonella enterica* complex, accounting for 51.72% of isolates, constitutes a substantial food safety issue that transcends regional limits, while the particular environmental and geographical conditions of Al-Baha may intensify these challenges. The significant incidence of *Salmonella* species in the Al-Baha study mirrors results from similar research. A meta-analysis examining the growth of *Salmonella* under refrigerated storage conditions revealed that *S.* Enteritidis displays remarkable resilience to cold storage, with documented survival at 4 °C and minimal inhibition of growth during extended refrigeration [37]. This corresponds precisely with the findings in the Al-Baha study, wherein *S.* Enteritidis represented the largest single isolated group, accounting for 27.59% of the total bacterial population. The persistence of *Salmonella* during refrigerated storage has been thoroughly documented in controlled studies. Previous research by Jiménez et al. [37] determined that the survival rates of *S.* Enteritidis were markedly affected by storage temperature, with samples stored at 5 °C exhibiting the smallest reduction in bacteria decline relative to higher temperatures. Likewise, an extensive investigation into the behavior of *Salmonella* in chicken meat during refrigerated storage demonstrated that adequate refrigeration at low temperatures inhibited bacterial proliferation, although complete eradication was not accomplished [38,39]. The observed 20.7% prevalence of *Escherichia* complex bacteria in this study signifies a pronounced level of fecal contamination and a lapse in hygiene protocols. This observation is consistent with extensive research which establishes *E. coli* as a dependable indicator organism for fecal contamination in poultry products [40,41].

The molecular strategic use of both anchored and non-anchored ISSR primers in this study enabled broad genomic coverage and improved discriminatory resolution among bacterial isolates [42,43]. Anchored primers such as HB13 and SAS1, which include additional 3′ nucleotides, exhibited high specificity and reliable amplification, yielding performance on par with established molecular typing tools [44]. These primers are known to reduce background noise and enhance target selectivity, a trend supported by studies on diverse bacterial taxa. Similarly, SAS1 displayed robust amplification and polymorphism detection, reinforcing its value in identifying closely related strains as a key requirement in epidemiological source tracking. This performance aligns with observations from plant genetics research, where anchored ISSR primers consistently yielded 80–95% polymorphism across species [45]. In contrast, the TERRY primer showed moderate efficacy, with occasional band smearing and reduced resolution among genetically similar isolates. Such variability is well-documented in ISSR-based studies, where primer efficiency often depends on genome complexity and microsatellite distribution. Among the non-anchored UBC series primers, UBC827 stood out as the most effective, offering exceptional discriminatory power and the highest information content. This result is consistent with prior research involving *Photorhabdus* and *Xenorhabdus* strains, where UBC primers exhibited 70–75% polymorphism [46]. Notably, UBC827 successfully differentiated isolates that other primers could not, highlighting its utility in outbreak investigation and source tracing. UBC809 and UBC811 also performed consistently well, achieving high-resolution separation of diverse bacterial taxa and detecting polymorphism rates exceeding 75%. Their broad amplification range (200–1500 bp) supports comprehensive genome coverage, reinforcing their effectiveness in phylogenetic studies and molecular epidemiology. While the use of ISSR markers in the identification of bacterial isolates presents a promising molecular approach that significantly improves the detection and differentiation of pathogenic strains [47]. When combined with other molecular techniques, ISSR enables rapid and precise identification of foodborne pathogens, which is critical for ensuring food safety and protecting public health. Integrating ISSR with PCR-based methods allows the amplification of specific genetic markers linked to common pathogens such as *E. coli* and *Campylobacter* species, frequently found in poultry products [48]. Notably, the combination of ISSR and real-time PCR has demonstrated high sensitivity and specificity, effectively distinguishing pathogenic *E. coli* strains, such as avian pathogenic *E. coli*, from their non-pathogenic counterparts [48]. Moreover, the joint application of ISSR and multiplex PCR has proven successful in detecting *Campylobacter jejuni* and *Campylobacter coli* two major contributors to foodborne diseases underscoring ISSR’s value as a reliable tool in microbial diagnostics and surveillance [49,50]. The single linkage dendrogram produced utilizing ISSR markers has demonstrated evident taxonomic consistency across various genetic distance thresholds [51]. At genetic distances of ≤5, all isolates of *Salmonella enterica* serovar Enteritidis (B2, B3, B5, B6, B7, B8) coalesced into a compact cluster, signifying a substantial degree of clonal similarity. Within a broader genetic distance range of 10–15, an intermediate assemblage materialized, comprising *S. Waycross* (B9, B11), *S.* Enteritidis (B13), *S. Minnesota* (B4), and *Escherichia* sp. (B10). This assemblage indicates moderate genetic variation within the Enterobacteriaceae family, congruent with findings from comparative genomic studies. Surpassing a genetic distance of 15, the dendrogram exhibited a distinct phylogenetic bifurcation, segregating Gram-positive genera such as *Bacillus anthracis* (B16) and *Arthrobacter* sp. (B20) from the Gram-negative Enterobacteriaceae. Significantly, *Citrobacter* youngae (B21) also assumed a discrete position, further underscoring the efficacy of ISSR markers in resolving both closely related taxa and more extensive phylogenetic distinctions.

The application of RAPD techniques has proven highly effective for the identification and characterization of bacterial isolates derived from poultry and food products [52]. These molecular tools significantly improve the precision of bacterial typing, which is vital for ensuring food safety and supporting epidemiological investigations. Specifically, RAPD-PCR has been extensively utilized to assess *Salmonella* spp. from poultry sources, successfully uncovering the genetic diversity among isolates. For example, one study analyzed 61 *Salmonella* isolates obtained from chickens and their surrounding environments, demonstrating RAPD’s strength in resolving genetic relationships among strains [53]. Similarly, another investigation employed RAPD to differentiate *Salmonella serovars* across various animal-origin food products, confirming the technique’s effectiveness in evaluating molecular heterogeneity [54]. These findings underscore RAPD’s practical value in monitoring bacterial contamination and supporting foodborne disease surveillance [55]. Therefore, in this study RAPD, OPA primer series exhibited varying degrees of discriminatory power, with each primer exhibiting specific strengths across phylogenetic scales. OPA08 provided high inter-genus resolution, generating 4–5 well-defined clusters within a similarity range of 0.45 to 0.95, rendering it particularly effective for differentiating between genera. OPA10 offered a balanced level of discrimination at both intra- and inter-species levels, making it ideally suited for epidemiological typing. Conversely, OPA18 produced more conservative groupings, prioritizing broad taxonomic separation over fine-scale resolution. Within the OPW series, OPW08 demonstrated superior capacity in detecting high levels of genetic heterogeneity, forming 5–6 distinct clusters with notable polymorphism, whereas OPW11 achieved a pragmatic balance for routine surveillance across various taxonomic levels. Notably, OPU15 distinguished itself through its exceptional strain-level resolution, differentiating isolates within a similarity range of 0.40 to 0.90, a performance comparable to whole-genome sequencing in certain contexts. The hierarchical clustering outcomes further validated the phylogenetic precision of RAPD markers. Clusters exhibiting ultra-high similarity (0–5) confirmed the clonal nature of *S.* Enteritidis isolates, whereas intermediate clusters (5–10) encapsulated evolutionary relationships among serovars. Moderate similarity groups (10–15) represented conserved relationships within the Enterobacteriaceae family, and clusters exhibiting maximal diversity (>20) distinctly separated Gram-positive from Gram-negative taxa. Collectively, these findings emphasize RAPD’s robustness and versatility for both fine-scale differentiation and broad taxonomic evaluation.

## 5. Conclusions

This molecular investigation of bacterial contamination in poultry refrigeration units within the Al-Baha region reveals critical food safety vulnerabilities that demand immediate attention from public health authorities and food industry stakeholders. The overwhelming predominance of Salmonella enterica complex (51.72% of isolates) represents a significant epidemiological concern, particularly given the exceptional resilience of *S.* Enteritidisto refrigeration temperatures and its documented ability to survive and maintain virulence at 4 °C storage conditions. The identification of multiple *Salmonella serovars*, including the highly pathogenic *S. enteritidis*, *S. typhimurium*, *S. dublin, S. minnesota*, and *S. waycross*, underscores the complexity of contamination sources and the potential for diverse transmission pathways within the regional food distribution network. The substantial presence of *Escherichia* complex bacteria (20.7%) serves as a clear indicator of fecal contamination and compromised hygiene protocols throughout the cold chain, from processing facilities to retail storage environments. The integrated application of ISSR and RAPD molecular markers represents a significant methodological advancement in bacterial strain characterization for food safety applications. This dual-marker approach demonstrated superior discriminatory capacity compared to single-marker systems, providing comprehensive coverage of genomic diversity while maintaining cost-effectiveness suitable for routine surveillance programs in developing regions. The exceptional performance of specific primers, particularly UBC827 in ISSR analysis and OPU15 in RAPD analysis, establishes a standardized molecular typing protocol with broad applicability across diverse bacterial taxa. The hierarchical clustering methodology successfully resolved phylogenetic relationships from clonal-level similarity to inter-genus differentiation, providing essential tools for outbreak investigation, source attribution, and epidemiological surveillance. The distinctive mountainous terrain and variable temperature conditions of Al-Baha compound the challenges associated with maintaining the integrity of the cold chain, thereby elevating the risk of microbial growth. These observations highlight the imperative for interventions that are specifically tailored to this region, including the formulation of customized hazard analysis and critical control points protocols that effectively address the local environmental limitations. The identification of antibiotic-resistant genera such as *Enterobacter cloacae* and *Citrobacter* spp. adds further complexity to the issue, raising significant concerns regarding the potential dissemination of antimicrobial resistance through foodborne pathways. The molecular framework established in this study facilitates focused surveillance, resistance monitoring, and source attribution. To fortify food safety measures, it is crucial to integrate obligatory molecular surveillance, advanced diagnostic tools providing real-time data, and genomic analyses. The study’s geographic limitation to Al-Mandaq in a single region, while restricting direct generalizability, strengthens its utility as a baseline for regional surveillance and provides a model for similar mountainous, resource-limited regions. Future investigations should employ: (1) longitudinal sampling (quarterly over ≥ 1 year), (2) expanded geographic coverage (other Al-Baha cities and Saudi regions), (3) subset WGS validation of representative isolates, and (4) metagenomic approaches to capture unculturable microbiota. Despite these limitations, the study successfully demonstrates the value of integrated molecular fingerprinting for strain-level bacterial discrimination in regional food safety surveillance, providing essential data to inform evidence-based interventions in Al-Baha’s poultry cold chain

## Figures and Tables

**Table 1:** List of ISSR primers used for ISSR amplification of bacterial isolates.

No.	Name of Primer	Sequence (5′→3′)
1	HB13	GAGGAGGAGGC
2	SAS1	GTGGTGGTGGTGGC
3	TERRY	GTGGTGGTGGGTGRC
4	UBC827	ACACACACACACACACG
5	UBC809	AGAGAGAGAGAG AGAGG
6	UBC811	GAGAGAGAGAGAGAGAC

**Table 2:** List of random primers used for RAPD amplification of bacterial isolates.

No.	Name of Primer	Sequence (5′→3′)
1	OPA-08	GTGACGTAGG
2	OPA-10	GTGATCGCAG
3	OPA-18	AGGTGACCGT
4	OPW-08	GACTGCCTCT
5	OPW-11	CTGATGCGTG
6	OPU-15	ACGGGCCAGT

**Table 3:** Bacterial strains were isolated from poultry refrigeration units in the Al-Baha region during the period 2–4 May 2024.

Number on the Gel	Supermarket ID	Closest Bacterial Strain from NCBI Blast	NCBI Submitted Accession Number	Ident. %
1	2	*Salmonella enterica* subsp. enterica serovar Waycross	PX021782	95.95
2	3	*Salmonella enterica* subsp. enterica serovar Enteritidis	PX021784	97.04
3	4	*Salmonella enterica* subsp. enterica serovar Enteritidis	PX021779	96.47
4	5	*Salmonella enterica* subsp. enterica serovar Minnesota	PX021775	100
5	7	*Salmonella enterica* subsp. enterica serovar Enteritidis	PX021772	91.42
6	10	*Salmonella enterica* subsp. enterica serovar Enteritidis	PX021787	91.97
7	17	*Salmonella enterica* subsp. enterica serovar Enteritidis	PX021776	97.81
8	20	*Salmonella enterica* subsp. enterica serovar Enteritidis	PX021774	95.99
9	27	*Salmonella enterica* subsp. enterica serovar Waycross	PX021786	96.76
10	29	*Escherichia* sp.	PX021773	99.91
11	30	*Salmonella enterica* subsp. enterica serovar Waycross	PX021767	96.99
12	35	*E. coli*	PX021785	100
13	40	*Salmonella enterica* subsp. enterica serovar Enteritidis	PX021792	96.38
14	41	*Escherichia coli*	PX021788	99.72
15	51	*Enterobacter cloacae*	PX021790	100
16	55	*Bacillus anthracis*	PX021791	99.47
17	59	*Salmonella enterica* subsp. enterica serovar Enteritidis	PX021777	100
18	63	*Escherichia fergusonii*	PX021771	99.82
19	69	*E. coli*	PX021770	99.56
20	72	*Arthrobacter* sp.	PX021768	99.91
21	78	*Citrobacter youngae*	PX021778	99.64
24	84	*Salmonella enterica* subsp. enterica serovar Typhimurium	PX021765	92.52
25	89	*Citrobacter* sp.	PX021793	99.47
30	99	*Bacillus anthracis*	PX021783	97.77
31	113	*Enterobacter* sp.	PX021781	100
33	119	*Bacillus* sp.	PX021780	96.51
34	120	*Salmonella enterica* subsp. enterica serovar Dublin	PX021766	95.78
36	133	*E. coli*	PX021769	99.29
38	142	*Salmonella enterica* subsp. enterica serovar Minnesota	PX021789	99.64

**Table 4:** Summary of ISSR and RAPD primer performance.

Primer	Total Bands	Polymorphic Bands	Polymorphism %
UBC827	22	20	90.9%
UBC809	19	17	89.5%
SAS1	18	16	88.9%
UBC811	17	15	88.2%
HB13	15	13	86.7%
TERRY	12	8	66.7%
OPW-08	20	17	85.0%
OPU-15	13	11	84.6%
OPA-10	18	15	83.3%
OPW-11	17	14	82.4%
OPA-08	16	13	81.2%
OPA-18	14	10	71.4%

## Data Availability

The original contributions presented in this study are included in the article material. Further inquiries can be directed to the corresponding authors.
